# Exophtalmie unilatérale révélant un géant anévrysme intra caverneux rompu de la carotide interne rompu

**DOI:** 10.11604/pamj.2019.33.104.16355

**Published:** 2019-06-11

**Authors:** Qariani Hajare, Aachak Meriem

**Affiliations:** 1 Hôpital Militaire d'Instruction Mohammed V, Rabat, Maroc

**Keywords:** Exophtalmie, paralysie du droit externe, anévrysme géant, sinus caverneux, Exophthalmos, paralysis of the right lateral rectus muscle, giant aneurysm, cavernous sinus

## Abstract

Giant aneurysm of the intracranial carotid artery is a relatively rare disorder which can lead to life-threatening consequences. Ophthalmologic symptoms, mainly oculomotor nerve palsies, usually reveal lesions of the cavernous sinuses. We report the case of a 39-year old female patient presenting with rapidly progressive unilateral exophthalmos (A, B) and binocular diplopia associated with headaches affect one half of the head causing a single episode of vomiting evolving in a context of apyrexia and general state preservation. Ophthalmologic examination showed isolated palsies of the right lateral rectus muscle (abduction deficit). Static and dynamic assessment of the eyelids was normal. Corrected visual acuity was 5/10. Slit lamp exam showed dilation of the conjunctival vessels (C), clear cornea that didn't take up fluorescein, preserved direct and consensual pupillary light reflex, intra-ocular pressure 15 mmhg and venous tortuosities at the back of the eye, without papillary abnormalities (D). Neuroradiological assessment, such as magnetic resonance angiography, showed compressive aneurysms of the intracavernous portion of the right internal carotid artery with grade III exophthalmos. Cerebral angiography objectified right giant ruptured saccular cavernous carotid aneurysm in the cavernous sinuses (E) with anterior and posterior venous drainage associated with aneurysm of the left carotid syphon (F).

## Image en médecine

L'anévrysme géant de la carotide intra-crânienne est une pathologie relativement rare et qui peut présenter des complications redoutables. Les lésions du sinus caverneux sont habituellement révélées par une symptomatologie ophtalmologique, principalement, les paralysies oculomotrices. Nous rapportons le cas d'une patiente de 39 ans qui présente une exophtalmie unilatérale d'installation rapidement progressive (A, B), et une diplopie binoculaire associées à des céphalées en hémi-casque avec un seul épisode de vomissements évoluant dans un contexte d'apyrexie et de conservation d'état général. L'examen ophtalmologique retrouve une paralysie isolée du droit externe droit (déficit d'abduction) avec un examen stato-dynamique des paupières normal. L'acuité visuelle corrigée est à 5/10. L'examen à la lampe à fente montre une dilatation des vaisseaux conjonctivaux (C), cornée claire ne prenant pas la fluorescéine, reflexe photomoteur direct et consensuel conservé, une pression intra-oculaire à 15 mmhg, et des tortuosités veineuses au fond d'œil sans anomalies papillaires (D). Le bilan neuroradiologique à savoir l'angio- imagerie à résonnance magnétique (IRM) révèle un anévrysme de la portion intra-caverneuse de la carotide interne droite, compressif, avec une exophtalmie stade 3. L'angiographie cérébrale est en faveur d'un anévrysme sacculaire géant carotido-caverneux droit rompu dans le sinus caverneux (E) avec drainage veineux antérieure et postérieure associé à un anévrisme du syphon carotidien gauche (F).

**Figure 1:**
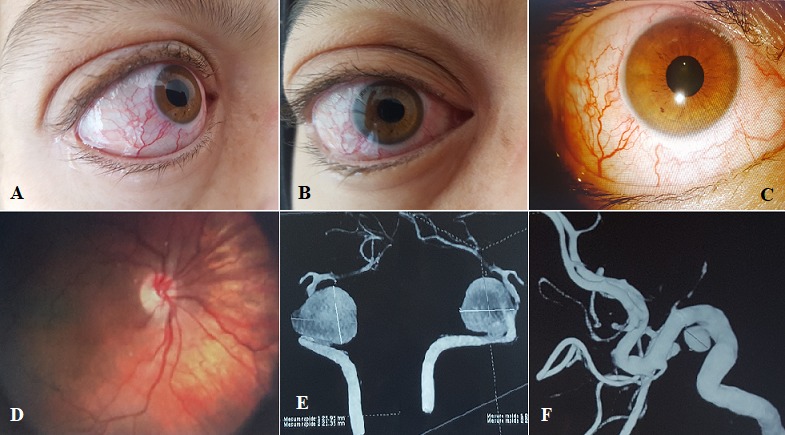
A) exophtalmie unilatérale droite (vue profile); B) exophtalmie unilatérale droite (vue de face); C) dilatation des vaisseaux conjonctivaux; D) tortuosité veineuse sans anomalie papillaire; E) anévrysme géant carotido-caverneux droit; F) anévrisme du syphon carotidien gauche

